# Metformin promotes tau aggregation and exacerbates abnormal behavior in a mouse model of tauopathy

**DOI:** 10.1186/s13024-016-0082-7

**Published:** 2016-02-09

**Authors:** Erica Barini, Odetta Antico, Yingjun Zhao, Francesco Asta, Valter Tucci, Tiziano Catelani, Roberto Marotta, Huaxi Xu, Laura Gasparini

**Affiliations:** Department of Neuroscience and Brain Technologies, Istituto Italiano di Tecnologia, Via Morego 30, Genoa, Italy; Fujian Provincial Key Laboratory of Neurodegenerative Disease and Aging Research, Institute of Neuroscience, College of Medicine, Xiamen University, Xiamen, Fujian 361102 China; Degenerative Diseases Program, Sanford Burnham Prebys Medical Discovery Institute, La Jolla, CA 92037 USA; Nanochemistry Department, Electron Microscopy Lab, Istituto Italiano di Tecnologia, Via Morego 30, Genoa, Italy; Present Address: AbbVie Deutschland GmbH &Co. KG, Knollstr., 67061 Ludwigshafen, Germany

**Keywords:** Alzheimer disease, Tau oligomers, Caspase 3, Cleaved-tau, Tau phosphorylation, Tau inclusions

## Abstract

**Background:**

Alzheimer disease (AD) and other tauopathies develop cerebral intracellular inclusions of hyperphosphorylated tau. Epidemiological and experimental evidence suggests a clear link between type 2 diabetes mellitus and AD. In AD animal models, tau pathology is exacerbated by metabolic comorbidities, such as insulin resistance and diabetes. Within this context, anitidiabetic drugs, including the widely-prescribed insulin-sensitizing drug metformin, are currently being investigated for AD therapy. However, their efficacy for tauopathy in vivo has not been tested.

**Results:**

Here, we report that in the P301S mutant human tau (P301S) transgenic mouse model of tauopathy, chronic administration of metformin exerts paradoxical effects on tau pathology. Despite reducing tau phosphorylation in the cortex and hippocampus via AMPK/mTOR and PP2A, metformin increases insoluble tau species (including tau oligomers) and the number of inclusions with β-sheet aggregates in the brain of P301S mice. In addition, metformin exacerbates hindlimb atrophy, increases P301S hyperactive behavior, induces tau cleavage by caspase 3 and disrupts synaptic structures.

**Conclusions:**

These findings indicate that metformin pro-aggregation effects mitigate the potential benefits arising from its dephosphorylating action, possibly leading to an overall increase of the risk of tauopathy in elderly diabetic patients.

**Electronic supplementary material:**

The online version of this article (doi:10.1186/s13024-016-0082-7) contains supplementary material, which is available to authorized users.

## Background

Intraneuronal inclusions made of filaments of hyperphosphorylated microtubule-associated tau protein are neuropathological hallmarks of a subset of neurodegenerative diseases termed tauopathies, a class that includes Alzheimer Disease (AD), corticobasal degeneration, progressive parasupranuclear palsy and frontotemporal dementia. In AD, tau inclusions co-exist with extracellular plaques of beta-amyloid (Aβ), another pathological hallmark of the disease. The levels of Aβ peptides and tau species, including total tau and phospho-tau, are altered in the cerebrospinal fluid (CSF) of subjects at risk for AD several years before putative onset of clinical symptoms [1]. However, only the severity of tauopathy strongly correlates with cognitive impairment in patients [2].

The progression of cerebral tau pathology appears to be influenced by several risk factors and comorbidities, including diabetes and insulin resistance. Patients with type-2 diabetes mellitus (T2DM) develop cognitive impairment and have an increased risk of developing AD (reviewed in refs. [3, 4]). Impaired cerebral glucose metabolism is also detected at early stages in AD [5] and insulin resistance occurs in the brain of some patients with AD independently of diabetes [6]. Further, molecular markers of insulin resistance co-localize with tau inclusions in AD brain [7], suggesting that impaired insulin signaling may be a key factor in the AD pathophysiological cascade. Considerable experimental and clinical data support this view: insulin signaling and insulin resistance affect the physiopathology of tau. Specifically, insulin transiently regulates tau phosphorylation in cultured neurons [8, 9], while reduced insulin signaling increases tau phosphorylation both in cultured neuronal cells [10] and in vivo models of insulin resistance and T2DM [4].

Within this context, the use of antidiabetic drugs has been proposed as potential therapy for AD [11]. Among these drugs, metformin is widely used for the treatment of T2DM, due to its safety profile and low cost. Albeit still controversial, there is experimental evidence that metformin may have beneficial effects on cognition. In rats fed with high-fat diet, metformin significantly attenuated insulin resistance and reverts the cognitive impairment induced by metabolic dysfunction [12]. However, other groups reported that metformin has no effect on specific cognitive tasks, such as behavioral flexibility, performance in the Barnes maze or fear conditioning in rodents with insulin resistance [13, 14]. Still, metformin improves cognitive function in experimental models unrelated to metabolic dysfunction such as the pentylentetrazole-induced kindling [15] and the haloperidol-induced catalepsy in adult mice [16], suggesting that some of its actions may be unrelated to its insulin-sensitizing properties. When applied for a few hours to neurons in vitro, metformin induces tau dephosphorylation either in basal condition [17] or after inducing insulin resistance [10]. However, in the db/db mouse model of insulin resistance, metformin dephosphorylates endogenous murine tau without affecting glycemia and metabolic parameters, suggesting that the effects on tau may be independent from the drug’s insulin-sensitizing action [14]. Nevertheless, it remains unknown whether in the absence of insulin resistance or diabetes, chronic treatment with metformin ameliorates tau pathology and behavioral performance in a transgenic model of neurodegenerative tauopathy in vivo.

To investigate this central question, we chronically administered metformin to the P301S mutant human tau transgenic (P301S) mouse, which develops tau inclusions throughout the central nervous system, neuronal loss [18, 19] and behavioral deficits [20]. Chronic metformin treatment reduces tau phosphorylation in the cortex and hippocampus of P301S mice by inducing protein phosphatase 2A (PP2A) expression via the AMPK/mTOR pathway. Paradoxically, metformin promotes the aggregation of recombinant tau in vitro and when given chronically to P301S mice, increases both the cortical amount of insoluble tau species (including tau oligomers) and the number of inclusions with β-sheet secondary structure. This dual action of metformin on tau pathology exacerbates neurodegeneration-induced hindlimb atrophy and hyperactive behavior in P301S mice. Metformin also activates caspase 3, increases tau cleavage by caspase 3 and disrupts synaptic structures. This work suggests that in subjects at risk for AD and tauopathy, metformin’s pro-aggregation effects may mitigate its beneficial actions on tau phosphorylation: caution is thus warranted for metformin application in chronic therapies in elderly diabetic patients.

## Results

### P301S mice show neither peripheral nor brain insulin resistance

**Fig. 1:**
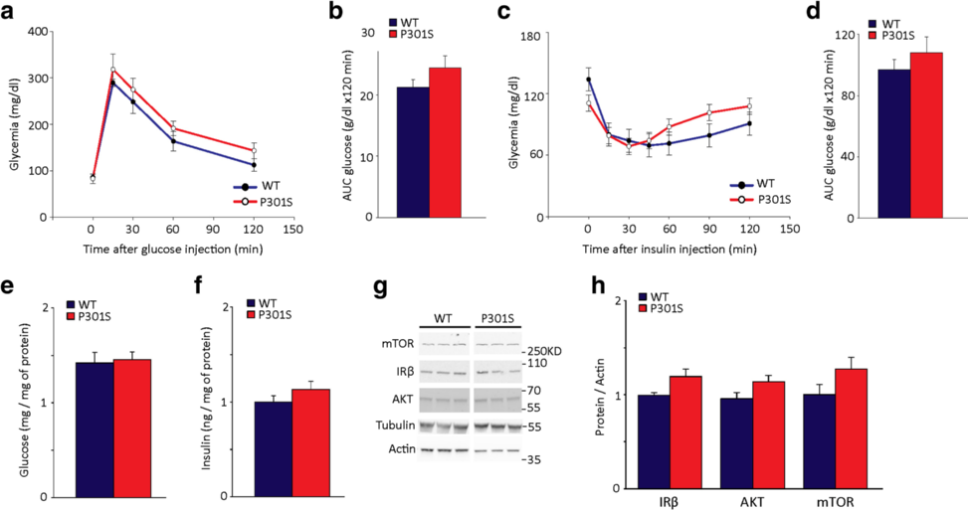
P301S mice show neither peripheral nor brain insulin resistance. **a** Intraperitoneal glucose tolerance test (IPGTT) in 5 month old WT non-transgenic and P301S transgenic mice. At time 0, *P* = 0.9; 15 min, *P* = 0.29; 30 min, *P* = 0.33; 60 min, *P* = 0.3; 120 min, *P* = 0.26; two-way ANOVA followed by Holm-Sidak multiple comparison test. P301S, *n* = 10; WT, *n* = 12. **b** IPGTT AUC. *P* = 0.17; Student *t*-test. **c** Insulin tolerance test (ITT) in 5 month old WT non-transgenic and P301S transgenic mice. At time 0, *P* = 0.09; 15 min, *P* = 0.95, 30 min, *P* = 0.69; 45 min, *P* = 0.73; 60 min, *P* = 0.24; 90 min, *P* = 0.11; 120 min, *P* = 0.22; two-way ANOVA followed by Holm-Sidak multiple comparison test. P301S, *n* = 12; WT, *n* = 6. In a and c, data points represent the average glycemia ± SEM. **d** ITT AUC. *P* = 0.50, Student *t*-test. **e**-**f** Glucose concentration (**e**) and insulin levels (**f**) in the cortex of 5-month old P301S and WT mice. *n* = 6 / group. Glucose and insulin concentrations were normalized to the sample protein content. Bars represent the average ± SEM. In panel e, *P* = 0.79; panel f, *P* = 0.24; Student *t*-test. **g** Western blot of mTOR, IRβ and Akt and total tau in the cortex. Actin and Tubulin were analyzed as a loading control. **h** Quantitative analysis of protein expression of IRβ (WT, *n* = 9; P301S, *n* = 12), AKT (WT, *n* = 8; P301S, *n* = 12) and mTOR (WT, *n* = 6; P301S, *n* = 7) in the cortex of 5-month old P301S and WT mice. Protein levels were normalized to actin. Data are expressed as percentage of WT. Bars represent the average ± SEM. IRβ, *P* = 0.06; AKT, *P* = 0.09; mTOR, *P* = 0.14; Student *t*-test

We first investigated whether brain tauopathy is associated with peripheral or central insulin resistance. We examined peripheral glucose homeostasis using the intra-peritoneal glucose tolerance test (IPGTT) and insulin tolerance test (ITT). For IPGTT, after fasting, mice were challenged with a glucose bolus and glycemia was monitored over 2 h. In both P301S transgenic and wild type (WT) non-transgenic mice, glycemia peaked within 15 min from the challenge, reached similar levels at all time points, and returned to baseline after 2 h (Fig. 1a), displaying a similar AUC for both transgenic and non-transgenic mice (Fig. 1b). In the ITT, in WT, glycemia quickly declined from the baseline of 134.2 ± 11.2 mg/dl to 80.0 ± 6.0 mg/dl 15 min after insulin injection and started recovering 60 min later, reaching values comparable to baseline after 2 h. In P301S and WT mice, glycemic values and AUC were similar (Fig. 1c, d).

To test brain insulin resistance, we measured cortical levels of glucose and insulin and analyzed the expression and activation of the insulin receptor (IR) and its downstream signaling molecules IR scaffold-1 (IRS1), protein kinase B (AKT) and mammalian target of rapamycin (mTOR). In the cortex of adult P301S and WT mice, levels of both glucose and insulin were similar (Fig. 1e, f). To assess IR expression, we evaluated its primary translation product IRβ at the mRNA level, and its protein precursor (IR precursor) and β subunit (IRβ). Levels of IRβ mRNA (Additional file 1: Figure S1A), IR precursor (Additional file 1: Figure S1B, C), and IRβ protein (Fig. 1g, h) were similar in P301S and age-matched WT mice. Cortical levels of both IRS1 and its form phosphorylated on Serine 616 (p[S616]IRS1), which indicates insulin resistance [6], were also similar in P301S and WT mice (Additional file 1: Figure S1B, D-E). Levels of AKT and mTOR and their active forms phosphorylated on Serine 473 (p[S473]AKT) and Serine 2448 (p[S2448]mTOR), respectively, were unchanged in P301S mice cortex (Fig. 1g, h; Additional file 1: Figure S1F-G). Signaling was similarly unchanged in the hippocampus. In both genotypes, there were comparable levels of hippocampal IRβ (IRβ / actin: WT, 1.0 ± 0.2; P301S, 1.5 ± 0.2; *P* = 0.15; Student *t*-test), IR precursor (IR precursor / actin: WT, 1.0 ± 0.1; P301S, 0.8 ± 0.1; *P* = 0.1, Student *t*-test), AKT (AKT / tubulin: WT, 1.0 ± 0.2; P301, 1.4 ± 0.2; *P* = 0.2, Student *t*-test), p[S473]AKT (p[S473]AKT / AKT: WT, 1.0 ± 0.1; P301S, 0.9 ± 0.05; *P* = 0.4, Student *t*-test), and mTOR (mTOR / tubulin: WT, 1.0 ± 0.2; P301S, 1.1 ± 0.2; *P* = 0.15; Student *t*-test).

Overall, these results indicate that in P301S mice, neither peripheral nor central insulin resistance associate with brain tauopathy.

### Metformin reduces tau phosphorylation in the brain of P301S mice

To investigate how metformin modulates tau pathology in vivo, we treated P301S mice with or without metformin for 4 months and analyzed levels of tau and its phosphorylation state in the cortex and hippocampus. Currently, metformin is prescribed for T2DM. Considering that diabetes diagnosis and treatment usually precede AD onset, we administered metformin according to a prevention scheme, starting the treatment at 4 weeks of age, when tauopathy is still minimal and asymptomatic in the P301S mouse [21]. The selected dose (2 mg/ml in the drinking water) achieved metformin plasma levels comparable to those obtained by therapeutic dosing in humans (Additional file 1: Table S1) [22] and in P301S transgenic mice, yielded brain levels comparable to those achieved in WT non transgenic mice (Additional file 1: Table S1).

**Fig. 2:**
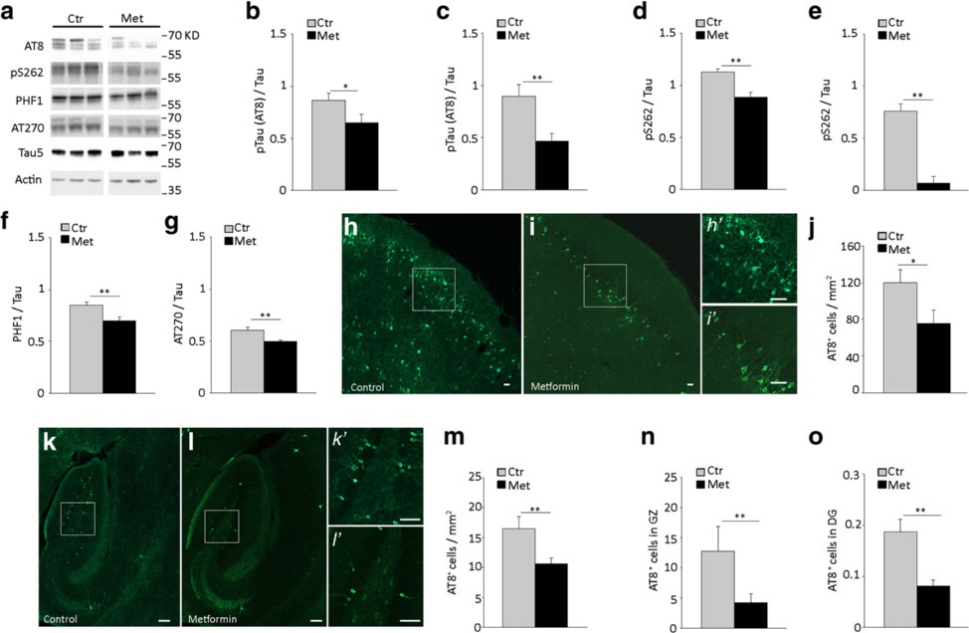
Metformin treatment reduces tau phosphorylation in vivo. Total tau and phosphorylated tau (pTau) were analyzed in the cortex and hippocampus of 5 month old P301S mice treated with or without metformin for 4 months. **a** Western blot of phosphorylated and total tau in the cortex. Actin was analyzed as a loading control. **b**-**g** Quantitative analysis of pTau in the cortex (**b**, **d**, **f**, **g**) and hippocampus (**c**, **e**). Phosphorylated tau was analyzed using AT8 (**b**, **c**), anti-phospho-S262 tau (**d**, **e**), PHF-1 (**f**), AT270 (**g**) antibodies and normalized to total tau levels. Total tau was analyzed using Tau5 antibody. Bars represent the average ratio over total tau ± SEM. *p* < 0.05, Student *t*-test. Panel b: n = 11 / group. Panel c: Ctr, *n* = 10; Met, *n* = 11. Panel d: Ctr, *n* = 12; Met, *n* = 12. Panel e: Ctr, *n* = 7; Met, *n* = 6; Panel f-g: Ctr, *n* = 7; Met, *n* = 7. **h**-**i** Representative fluorescence images of AT8 immunoreactivity in the cortex of untreated and metformin-treated P301S mice. Scale bars: in H-I, 50 μm; in *h’*-*i’*, 150 μm. Panels *h’* and *i’* are magnification of squared areas in panels h and i. **j**. AT8 positive (AT8^+^) cells in the prefrontal cortex. **k**-**l** Representative fluorescence images of AT8 immunoreactivity in hippocampi of untreated and metformin-treated P301S mice. Scale bars: in k-l, 100 μm; in *k’*-*l’*, 200 μm. Panels *k’* and *l’* are magnification of squared areas in panels k and l, respectively. **m**-**o** AT8 positive (AT8^+^) cells in the hippocampus (**m**), granular zone (**n**) and dentate gyrus (**o**). Panel j: Ctr, *n* = 3; Met, *n* = 4; Panel m-o: Ctr, *n* = 3; Met, *n* = 5. In j and m-o, bars represent the average cell number / mm^2^ ± SEM. *p < 0.05, **p < 0.01, Student *t*-test

Total tau protein levels were similar in P301S mice with or without metformin, both in the cortex (Tau5 / actin: Ctr, 1.0 ± 0.17; Met, 1.03 ± 0.2. *P* = 0.9, Student *t*-test) and hippocampus (Ctr, 1.0 ± 0.1; Met, 1.3 ± 0.1; *P* = 0.15, Student *t*-test). However, tau phosphorylation was significantly reduced in metformin-treated P301S mice. Levels of tau phosphorylated on Serine 262 and the phospho-epitopes recognized by AT8 antibody were significantly decreased in both the cortex (Fig. 2a-b, d) and hippocampus (Fig. 2c, e). In the cortex of metformin-treated P301S mice, phosphorylation on epitopes recognized by PHF1 and AT270 was also decreased (Fig. 2f-g).

In 5-month old P301S mice, phosphorylated tau accumulates in some, but not all neurons throughout the brain [18], reaching different degrees of tau pathology in distinct brain areas. Consistent with this variation, the number of AT8 positive cells is higher in the prefrontal cortex than the hippocampus of 5-month old P301S mice (Fig. 2h, j, k, m). To investigate whether metformin treatment changes the number of tauopathy-affected neurons, we counted AT8 immunoreactive (AT8^+^) cells in the hippocampus and prefrontal cortex of transgenic mice treated with or without metformin. Consistent with biochemical findings, in P301S mice treated with metformin, the number of AT8^+^ neurons was significantly reduced in both the prefrontal cortex (Fig. 2h-j) and hippocampus (Fig. 2k-m). Specifically, in the hippocampus, the number of AT8^+^ cells was reduced by 67 % in the granular zone (Fig. 2n), which contains the majority of hippocampal AT8^+^ cells, and by 57 % in the dentate gyrus (Fig. 2o), but was unchanged in the subiculum (AT8^+^ cells / 10 mm^2^: Ctr, 0.2 ± 0.03; Met, 0.1 ± 0.01; *P* = 0.1, Student *t*-test).

These results indicate that in P301S mice, chronic treatment with metformin decreases the phosphorylation of tau on several epitopes and reduces the number of neurons accumulating phospho-tau in the cortex and hippocampus.

### Metformin increases PP2A expression in the brain of P301S mice

**Fig. 3:**
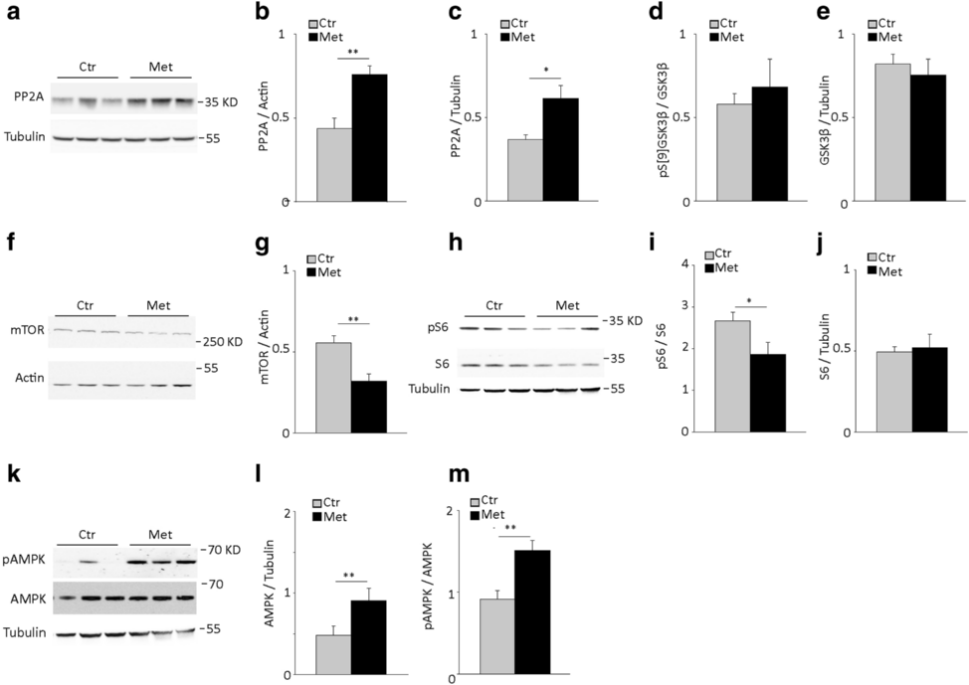
Metformin treatment induces PP2A expression, inhibits mTOR and activates AMPK in the brain of P301S mice. Protein levels of PP2A, GSK3β, pGSK3β, mTOR, S6, phospho-S6 (pS6), AMPK and phospho-AMPK (pAMPK) were analyzed by western blot in the brain of 5 month old P301S mice treated with or without metformin for 4 months. **a** Western blot of PP2A in the cortex. Tubulin was analyzed as a loading control. **b**-**c** Quantitative analysis of PP2A in the cortex (**b**) and hippocampus (**c**). In panel b: Ctr, *n* = 10; Met, *n* = 12. In panel c: Ctr, *n* = 9; Met, *n* = 11. **d**-**e** Quantitative analysis of p[S9]GSK3β (**d**) and GSK3β (**e**) in the cortex. Ctr, *n* = 13; Met, *n* = 12. **f** Western blot of mTOR in the cortex. Actin was analyzed as a loading control. **g** Quantitative analysis of mTOR protein expression. Ctr, *n* = 7; Met, *n* = 7. **h** Western blot of pS6 and S6 in the cortex. Tubulin was analyzed as a loading control. **i**-**j** Quantitative analysis of pS6 (**i**) and S6 (j). *n* = 7 / group. **k** Western blot of pAMPK and AMPK in the cortex. Tubulin was analyzed as a loading control. **l**-**m** Quantitative analysis of AMPK (**l**) and pAMPK (**m**). Panel l-m: Ctr, *n* = 10; Met, *n* = 12. Protein expression levels were normalized to tubulin or actin as indicated in the graphs. Levels of phosphorylated proteins were normalized to the total respective protein. In all graphs, bars represent the average ratio ± SEM. *p < 0.05, **p < 0.01, Student *t*-test

Physiological phosphorylation of tau depends on the balance between protein kinase and phosphatase activity. Protein phosphatase 2A (PP2A) is the main tau-targeting protein phosphatase [23], while among kinases, glycogen synthase kinase 3β (GSK3β) plays a key role in insulin-regulated tau phosphorylation [24]. Metformin acts on both PP2A [17] and GSK3β [10] in cultured neuronal cells. To investigate how metformin reduces tau phosphorylation in vivo, we analyzed the protein and mRNA expression of PP2A and the protein level and activation of GSK3β. After metformin treatment, PP2A protein expression was significantly increased in both cortex and hippocampus of P301S mice (Fig. 3a-c). Specifically, in the cortex, metformin increased PP2A protein expression by 60 % over untreated transgenic mice (Fig. 3b). To test whether metformin acts at the level of transcription, we analyzed PP2A mRNA. In P301S mice, metformin treatment significantly increased PP2A mRNA in both cortex (Ctr: 0.69 ± 0.09; Met 1.2 ± 0.15, *P* = 0.01, Student *t*-test) and hippocampus (Ctr: 0.96 ± 0.08; Met: 1.25 ± 0.02; *P* = 0.01; Student *t*-test). We next investigated GSK3β and its phosphorylation on Serine 9 (p[S9]GSK3β), which inhibits GSK3β [25]. Chronic treatment with metformin alters neither GSK3β protein expression nor p[S9]GSK3β levels (Fig. 3d-e), indicating that metformin does not inhibit GSK3β activity.

Overall, these findings indicate that in P301S transgenic mice, chronic treatment with metformin significantly enhances PP2A expression at mRNA and protein levels, without changing overall GSK3β activity.

### Metformin activates the AMPK-mTOR pathway in the brain of P301S mice

PP2A is regulated by the integration of several intracellular signals, including the insulin signaling pathway [26] and/or inhibition of mTOR through phosphorylation by AMPK [27, 28]. We therefore investigated whether chronic metformin activates any of these pathways in the brain of P301S mice.

We first analyzed the expression and activation of AMPK and mTOR. Chronic administration of metformin significantly reduced by 43 % mTOR levels in the cortex of P301S mice (Fig. 3f-g). To examine mTOR activity, we analyzed the total levels and phosphorylation of S6 (Fig. 3h-j), the substrate of the mTOR downstream target protein kinase S6 [29]. Treatment with metformin significantly reduced by 23 % the levels of phospho-S6 (Fig. 3i) without affecting S6 expression (Fig. 3j), indicating that metformin inhibits mTOR signaling in the brain of P301S mice.

mTOR is inhibited by AKT phosphorylation on serine 2448 (p[S2448]mTOR) [30] via the insulin signaling pathway and by AMPK phosphorylation on threonine 2446 [31]. To dissect the mechanisms underlying mTOR inhibition, we therefore analyzed the insulin signaling pathway, AMPK and its activation. We first examined levels of p[S2448]mTOR, IRβ, IRS1, AKT and its phosphorylated active form (pAKT). There were no significant differences in the levels of these signaling molecules in metformin-treated P301S mice (Additional file 1: Figure S2A-E), arguing against a direct involvement of insulin signaling in metformin’s effects on mTOR.

We next investigated AMPK expression levels and phosphorylation on threonine 172 (p[T172]AMPK), which indicates its activation state [32]. In P301S mice, chronic treatment with metformin slightly, but significantly increased AMPK protein expression (Fig. 3l) and strongly enhanced its activation (Fig. 3m).

Together, these results suggest that chronic administration of metformin inhibits mTOR and activates AMPK in the brain of P301S mice, without affecting insulin signaling.

### Metformin reduces tau phosphorylation through activation of PP2A via AMPK/mTOR in vitro

**Fig. 4:**
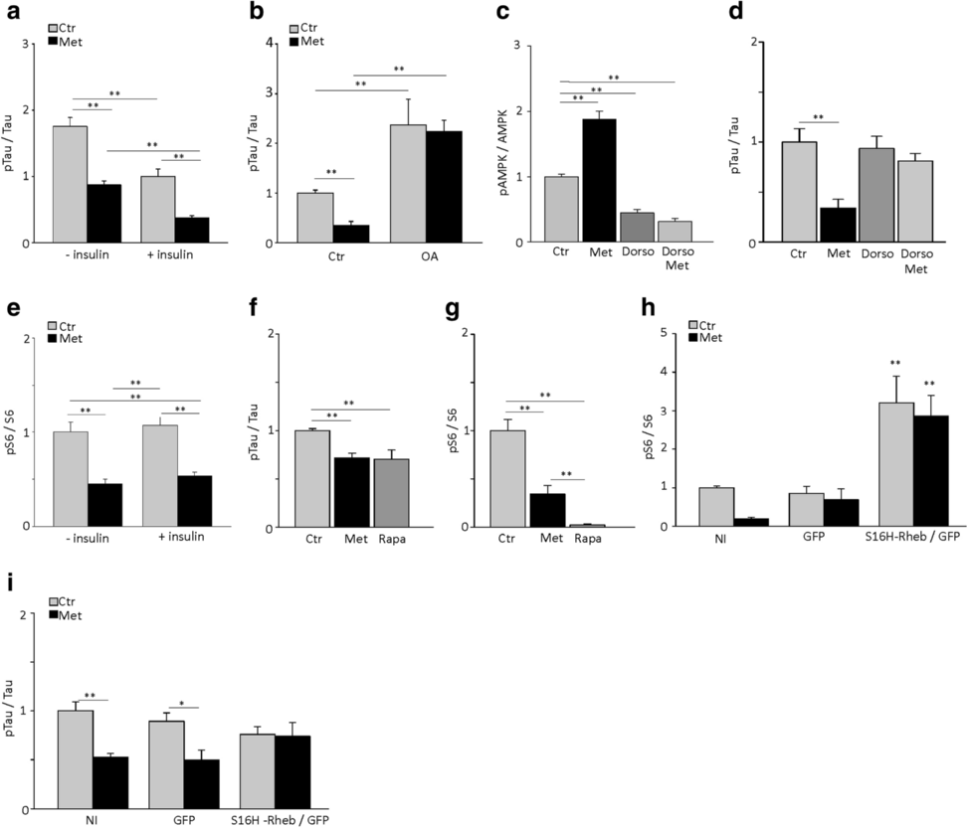
Metformin reduces tau phosphorylation in mouse cortical neurons via the AMPK-mTOR pathway. Primary cortical neurons were incubated for 6 h with or without 2.5 mM metformin (Met), 10 nM okadaic acid (OA), 10 μM rapamycin (Rapa), and 10 μM dorsomorphin (Dorso) alone or in combination. All treatments were performed in the absence of insulin, except when specifically indicated. **a** Quantitative analysis of phospho-tau (pTau) in cortical neurons treated with or without metformin in the absence or presence of insulin. **b** Quantitative analysis of pTau in cortical neurons treated with or without metformin in the absence or presence of okadaic acid. **c**-**d** Quantitative analysis of pAMPK (**c**) and pTau (**d**) in cortical neurons treated with or without metformin and dorsomorphin alone or in combination. **e** Quantitative analysis of pS6 in cortical neurons treated with or without metformin in the absence or presence of insulin. **f**-**g**. Quantitative analysis of pTau (**f**) and pS6 (**g**) in cortical neurons treated with or without metformin and rapamycin. **h**-**i**. Quantitative analysis of pS6 (**h**) and pTau (**i**) in neurons non-infected (NI), infected with GFP and S16H-Rheb or GFP alone and treated with metformin. Levels of phosphorylated protein were normalized to levels of respective proteins. In panels a, b, d, f, and i, pTau and total tau were analyzed using AT8 and Tau5 antibodies, respectively. In all graphs, bars represent the average ratio ± SEM of 3 independent experiments. *p < 0.05, **p < 0.01, one-way ANOVA followed by Holm-Sidak multiple comparison test

Our results indicate that in the brain of P301S mice, metformin increases the expression and activation of AMPK, inhibits mTOR and induces PP2A expression. To investigate whether these signaling and effector molecules mediate the effects of metformin on tau phosphorylation, we incubated primary cortical neurons with or without metformin, alone or in combination with specific inhibitors or activators and analyzed tau phosphorylation. Consistent with previous findings [17], incubation with metformin alone significantly reduced tau phosphorylation (Fig. 4a) without affecting tau expression levels (Additional file 1: Figure S3A). The effects of metformin were independent from insulin. Despite that levels of phospho-tau were significantly higher in neurons cultured in the absence of insulin than in those cultured with insulin, metformin reduced tau phosphorylation similarly in both cases (Fig. 4a).

To examine PP2A involvement, we treated cortical neurons with metformin alone or in combination with the PP2A inhibitor, okadaic acid (OA). As previously reported [17], OA alone increased the levels of tau phosphorylation (Fig. 4b), without changing total tau expression (tau / Tubulin. Ctr: 1.0 ± 0.08; Met: 0.97 ± 0.4, OA: 0.7 ± 0.08, OA + Met: 0.7 ± 0.06. *P* = 0.6, One Way ANOVA). When co-incubated, OA abolished metformin-induced tau dephosphorylation in primary neurons (Fig. 4b) indicating that metformin’s effect on tau phosphorylation requires PP2A activity.

We next investigated whether AMPK and mTOR activity were necessary for metformin’s action on tau phosphorylation. For AMPK, we incubated cortical neurons with metformin alone or in combination with 10 μM dorsomorphin, an inhibitor of AMPK activity in vitro [33]. Dorsomorphin strongly inhibited AMPK as judged by reduced levels of p[T172]AMPK (Fig. 4c) and completely prevented metformin-induced tau dephosphorylation (Fig. 4d), without changing expression levels of AMPK (Additional file 1: Figure S3B). In neurons treated with metformin, mTOR was inhibited in the presence and absence of insulin (Fig. 4e). To examine the role of mTOR in metformin’s action on tau phosphorylation, we inhibited its activity with rapamycin, or activated it by overexpressing a constitutive active mutant of the mTOR activator Rheb (S16H-Rheb) [34]. Both metformin and rapamycin reduced tau phosphorylation (Fig. 4f) and inhibited mTOR, as indicated by decreased phospho-S6 levels (Fig. 4g), without affecting S6 protein expression (Additional file 1: Figure S3C). To investigate whether mTOR is specifically required for metformin-induced tau dephosphorylation, we transduced primary cortical neurons with lentiviral particles containing the GFP reporter along with constitutively active S16H-Rheb. S16H-Rheb activated mTOR in primary cortical neurons as judged by increased levels of phospho-S6 (Additional file 1: Figure S3E, Fig. 4h), leaving unchanged S6 expression (Additional file 1: Figure S3F). S16H-Rheb expression abolished metformin-induced tau dephosphorylation (Fig. 4i), without affecting tau expression levels (Additional file 1: Figure S3G).

Overall, these results indicate that the effects of metformin on tau phosphorylation occur in an insulin-independent manner and require the activity of AMPK and PP2A and inhibition of mTOR.

### Metformin increases insoluble tau and tau inclusions in the P301S brain and promotes tau oligomeric aggregation in vitro

**Fig. 5:**
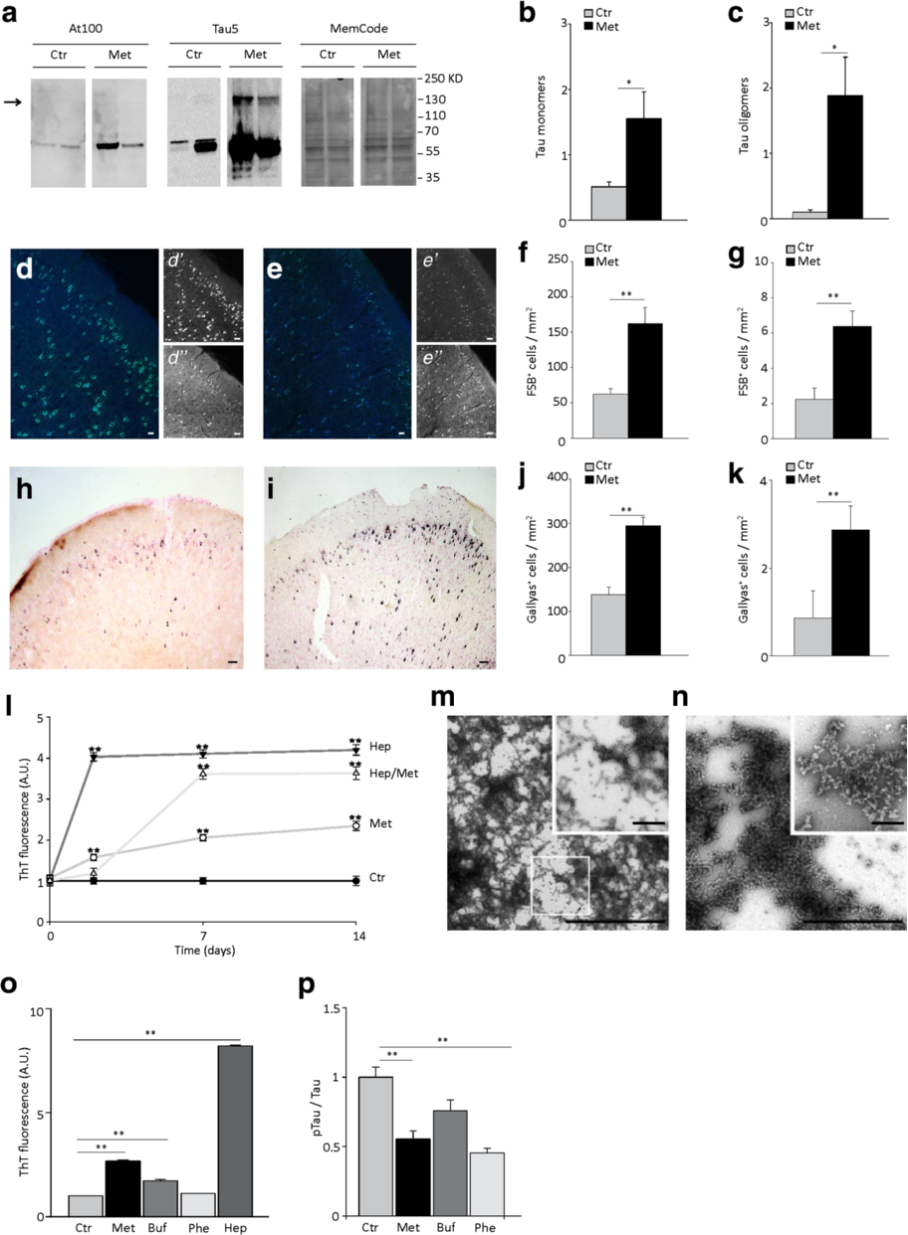
Metformin increases the number of tau inclusions in P301S mouse brain in vivo and induces aggregation of recombinant P301S tau in vitro. **a** Western blot analysis of tau in the sarkosyl-insoluble cortical fraction from P301S mice treated with or without metformin. The arrow indicates the bands of tau dimers. Tau was detected using the phosphorylation-and conformation dependent AT100 and Tau5 anti-tau monoclonal antibodies. MemCode was used to control equal loading. **b**-**c** Quantitative analysis of monomeric (**b**) and dimeric (**c**) tau in sarkosyl-insoluble fractions of P301S cortex. Densitometric values of tau bands are normalized over MemCode and bars represent the average ratio ± SEM. P301S, *n* = 4 / group. **d**. Representative images of MC1 immunoreactivity (**d**, d’; green) and FSB (**d**, d”; light blue) fluorescence in the cortex of P301S mice. Scale bars: in **d**, 50 μm; in *d’*-*d”*, 100 μm. **e** Representative images of AT8 immunoreactivity (e, e’; green) and FSB (e, e”; light blue) fluorescence in the cortex of P301S mice. Scale bars: in **e**, 50 μm; in *e’*-*e”*, 150 μm. **f**-**g** FSB^+^ cells in the prefrontal cortex (**f**) and hippocampus (**g**) of P301S mice. *n* = 3 / group. Bars represent the average number of FSB^+^ cells / mm^2^ ± SEM. **p < 0.01, Student *t*-test. **h**-**i**. Gallyas silver staining in the cortex of P301S mice untreated (**h**) or treated with metformin (**i**). Scale bar 150 μm. **j**-**k**. Gallyas^+^ cells in the prefrontal cortex (**j**) and hippocampus (**k**) of P301S mice. *n* = 3 / group. Bars represent the average number of Gallyas^+^ cells / mm^2^ ± SEM. **p < 0.01, Student *t*-test. **l**. Thioflavin T (ThT) fluorescence of recombinant P301S mutant human tau (P301Stau) aggregated in vitro in the absence (Ctr) or presence of 37.5 μM heparin (Hep) and/or 30 μM metformin (Met) for 2, 7 and 14 days. Data points represent the average ± SEM from at least 3 independent experiments. **m**-**n**. Representative negative staining electron microscopy image of fibrils of recombinant P301Stau aggregated in vitro for 7 days in presence of heparin (**m**) or metformin (**n**). In **m**, inset shows a high magnification of the squared area. In **n**, inset shows a high magnification image of tau aggregates. Scale bars: i-j, 2.5 μm; insets, 0.5 μm. **o** ThT fluorescence of recombinant P301Stau aggregated in vitro in the absence (control, Ctr) or presence of 37.5 μM heparin, 30 μM metformin, 30 μM phenformin (Phe) or 30 μM buformin (Buf) for 7 days. Fluorescence values are expressed as arbitrary units and normalized to the control. Bars represent the average ± SEM. **p < 0.01, one-way ANOVA followed by Holm-Sidak multiple comparison test. **p** Quantitative analysis of pTau in cortical neurons treated with or without 2.5 mM metformin, 30 μM phenformin and 30 μM buformin. pTau was detected using the AT8 monoclonal antibody and normalized on total tau detected by Tau5 antibody. Data are expressed as percentage of Ctr. Bars represent the average percentage ± SEM from 2 independent experiments. *p < 0.05, **p < 0.01, one-way ANOVA followed by Holm-Sidak multiple comparison test

We next investigated whether reducing tau phosphorylation translates into amelioration of tau filamentous inclusions. We first isolated insoluble tau filaments from the cortex of 5-month old P301S mice treated with or without metformin using the sarkosyl extraction. In the sarkosyl-insoluble fraction of P301S cortex, western blot analysis using Tau5 antibody revealed major monomeric tau bands at ≈ 58-60 kDa, a smear at higher molecular weights with a clear double band at ≈ 115–120 kDa, which is consistent with the molecular weight of tau dimers. Bands of monomeric tau were also detected by the AT100 antibody, which recognized conformations typical of tau filamentous inclusions [35]. The amount of insoluble tau, both monomeric and oligomeric, was significantly increased in metformin-treated P301S mice, with augmented levels of tau monomers (Fig. 5a-b), dimers (Fig. 5a, c) and AT100 immunoreactivity (Fig. 5a). We next analyzed cerebral tau inclusions by immunohistochemistry. Brain sections were immunostained with the conformation-dependent anti-tau antibody MC1 [36] or anti-phospho-tau AT8 antibody and counterstained with the Congo red analogue FSB. All FSB positive (FSB^+^) neurons were immunoreactive for MC1 antibody (Fig. 5d), but only some of them were labeled by AT8 (Fig. 5e). We next counted the FSB^+^ cells in the prefrontal cortex and hippocampus of P301S mice, chronically treated with metformin or untreated. In 5-month old P301S mice, FSB^+^ inclusions were more abundant in the prefrontal cortex (62.2 ± 7.7 inclusions / mm^2^; Fig. 5f) than in the hippocampus (2.2 ± 0.6 inclusions / mm^2^; Fig. 5g). When P301S mice were treated with metformin, the number of FSB^+^ inclusions significantly increased by 2.5- and 2.8-fold in the prefrontal cortex (Fig. 5f) and hippocampus (Fig. 5g), respectively. Similar results were obtained using Gallyas silver staining (Fig. 5h-k). The number of Gallyas + cells was significantly increased in the prefrontal cortex (Fig. 5h, j) and hippocampus (Fig. 5k) of P301S mice treated with metformin. Therefore, despite reducing tau phosphorylation, chronic treatment with metformin significantly increases the formation of insoluble aggregates with β-sheet secondary structure in vivo.

We hypothesized that metformin could increase tau inclusions through direct effects on tau aggregation. To test this, we evaluated whether metformin promotes aggregation of recombinant P301S mutant human tau (tauP301S) in vitro using an established heparin-induced aggregation protocol and the thioflavin T assay. When incubated in vitro with heparin, recombinant tauP301S aggregated into fibrils, as detected by both thioflavin T assay and negative staining transmission electron microscopy (Fig. 5l-n). The aggregation process was time-dependent and quickly reached maximal fluorescence values (Fig. 5l) due to abundant tau fibrils (Fig. 5l). We then performed the assay substituting heparin with metformin or incubating recombinant tauP301S with both heparin and metformin. With metformin alone, recombinant tauP301S aggregates in β-sheet-containing species in a time-dependent manner (Fig. 5l), reaching fluorescence values equal to half of those obtained with heparin after 14 days in vitro. When incubated with both metformin and heparin, there was an increased aggregation lag-time and fluorescence levels were intermediate between those found with metformin or heparin alone (Fig. 5h). At negative staining TEM, aggregates obtained with metformin alone had a different structure compared to those obtained with heparin after 7 days of incubation (Fig. 5m-n). With heparin, tau forms abundant filaments (Fig. 5m). In contrast in presence of metformin, we observed branched aggregates, formed by sub-spherical units, with a diameter of around 40 nm, aligned in beaded chains (Fig. 5n). These structures resembled tau protofibrils, formed by aligned spherical nucleation units (SNUs), previously described as precursors of tau filaments [37].

We next investigated whether other members of the biguanide class of antidiabetic drugs with different chemical structure, i.e., phenformin and buformin, share the pro-aggregation effect of metformin. After 7 days of incubation, buformin, but not phenformin, promoted tau aggregation in vitro (Fig. 5o). There were fewer aggregates with buformin than with metformin (Fig. 5o). These results indicate that both metformin and to some extent buformin act as pro-aggregants on recombinant tau in vitro.

Considering the possibility that metformin may interact directly with tau, we postulated that the paradoxical effect on tau phosphorylation could be an artifact due to an interaction masking the epitope. To test this hypothesis, we spiked cortical lysates from 5-month old P301S mice with increasing concentrations (2.5–250 μM) of metformin and analyzed phospho-tau by western blot with the AT8 antibody. The amount of phospho-tau detected was similar in all samples, irrespective of metformin concentration (Ctr, 1 ± 0.001; 2.5 μM Met, 1.4 ± 0.1; 25 μM Met, 0.8 ± 0.07; 250 μM Met, 1 ± 0.1; *P* = 0.06, one-way ANOVA), indicating that potential tau interaction and epitope masking do not cause the effects of metformin on phosphorylation.

Finally, we investigated whether buformin and phenformin also affect tau phosphorylation. Due to toxicity, these biguanides could not be used at concentration equimolar with metformin and were applied to primary neurons at 30 μM. At this concentration, phenformin significantly reduced tau phosphorylation in primary cortical neurons, while buformin did not have any effect on tau phosphorylation levels (Fig. 5p).

In summary, metformin concurrently induces tau dephosphorylation and promotes its aggregation, while other biguanides retain only one or the other of these activities.

### Metformin treatment enhances hyperactive behavior in the open field test

Since metformin treatment shows a dual action on tauopathy, by reducing tau phosphorylation and increasing formation of aggregates with β-sheet secondary structure, we investigated whether the drug’s effects affected the behavioral performance in the open field (OF) task.

**Figure 6:**
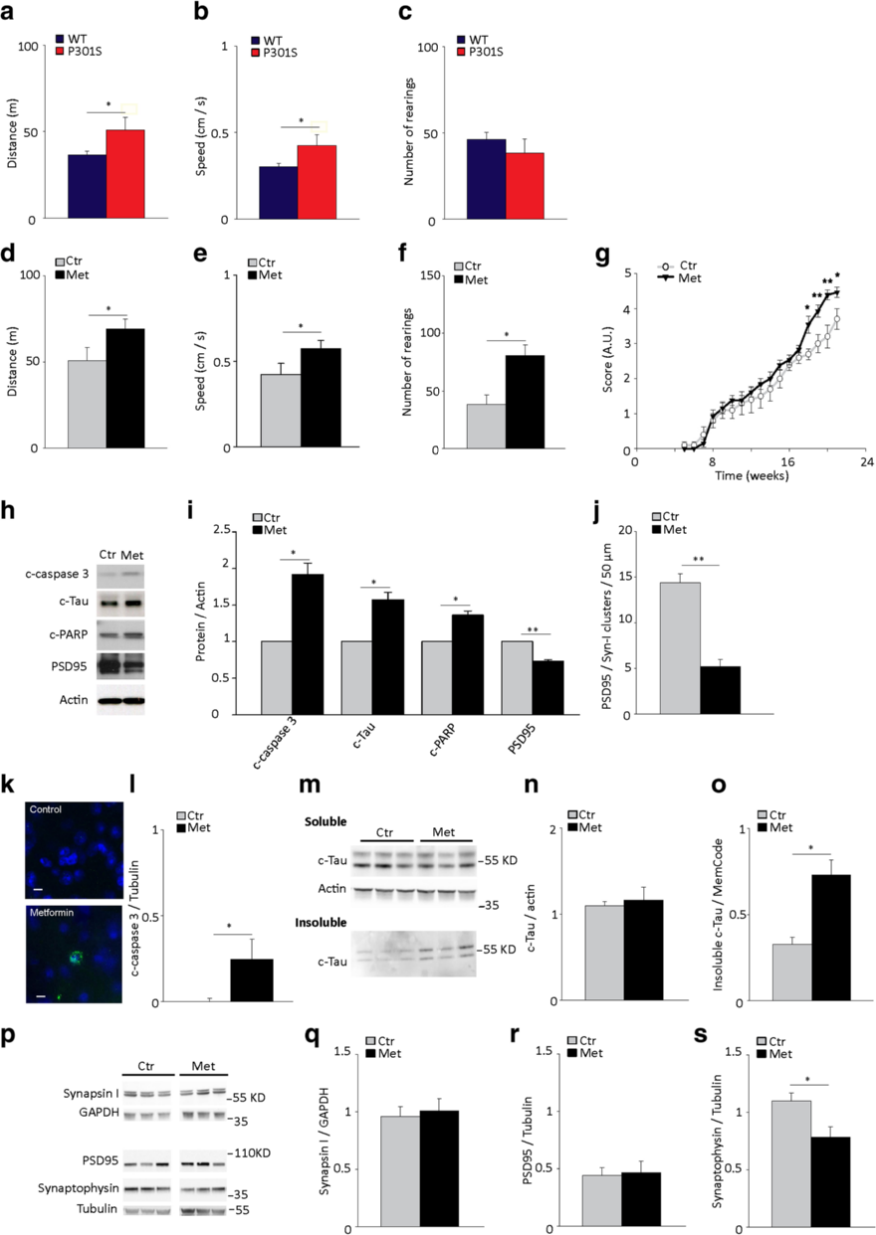
Metformin worsens hindlimb atrophy and hyperactive behavior and induces caspase 3 activation and tau cleavage. **a**-**c** Open field test in 5-month old P301S transgenic mice and age-matched WT non transgenic mice. Distance travelled (**a**), speed (**b**) and number of rearings (**c**) are shown. Bars represent the average values ± SEM. *p < 0.05, Student *t*-test. *n* = 9 / group. **d**-**g**, **k**-**s**. P301S mice were treated with or without metformin (Met) for 4 months starting from 1 month of age. **d**-**f**. Open field test. Distance travelled (**d**), speed (**e**) and rearing (**f**) are shown. Bars represent the average values ± SEM. *p < 0.05, Student *t*-test. Panels d-f: Ctr, *n* = 11; Met, *n* = 9. **g** Hind limb extension test in P301S mice treated with or without metformin. Data points represent the average score ± SEM. *p < 0.05, two-way ANOVA followed by Holm-Sidak multiple comparison test. A.U., arbitrary units. Ctr, *n* = 12; Met, *n* = 13. **h**-**j**. Primary cortical neurons were treated with or without 0.5 mM metformin for 24 h. **h**-**i**. Western blot (**h**) and quantitative analyses (**i**) of cleaved caspase 3 (c-caspase 3), c-Tau, c-PARP and PSD95. Protein levels were normalized to actin. Bars represent the average values ± SEM. *p < 0.05, **p < 0.01, Student *t*-test. *n* = 3/group. **j**. Quantification of clusters with overlapping synapsin-I and PSD95 immunoreactivity. Bars represent the average ± SEM. **p < 0.01, Student *t*-test. *n* = 11/group. **k** Representative confocal maximal projection images of active c-caspase 3 immunoreactivity (*green*) in the prefrontal cortex of untreated and metformin-treated P301S mice. Nuclei are counterstained with Hoechst 33342. Scale bar 10 μm. **l** Quantitative analysis of active c-caspase 3. c-caspase 3 was analyzed by western blot and protein levels were normalized to tubulin. Bars represent the average ± SEM. *n* = 7 / group. *p < 0.05, Student *t*-test. **m** Western blot analysis of cleaved-tau (c-Tau) in the soluble and sarkosyl-insoluble fraction of metformin-treated and untreated P301S mouse cortex. **n**-**o** Quantitative analysis of cleaved Tau (c-Tau) in the soluble (**n**) and insoluble (**o**) cortical fraction. c-Tau was analyzed by western blot and protein levels were normalized to actin (**n**) or MemCode (**o**). Bars represent the average ± SEM. Panel n: *n* = 7 / group. Panel o: *n* = 3/group. **p** Western blot analysis of synapsin I, PSD95, synaptophysin, tubulin and GAPDH in the cortex of P301S mice treated with or without metformin. **q**-**s**. Quantitative analysis of synapsin I (**q**), PSD95 (**r**) and synaptophysin (**s**). Protein levels were analyzed by western bot and normalized to tubulin or GAPDH. Bars represent the average ± SEM. *p < 0.05, Student *t*-test. Panel q: *n* = 7 / group. Panel r: Ctr, *n* = 13; Met, *n* = 10. Panel s: Ctr, *n* = 13; Met, *n* = 11

In the OF, P301S mice display a hyperactive behavior (i.e., increased distance travelled and speed) without changes of rearing behavior [20]. Consistently, we found that 5 month old P301S mice travel an increased distance, move at higher speed in the OF (Fig. 6a-b) than WT mice, but have preserved rearing behavior (Fig. 6c). The hyperactive behavior in the OF has been ascribed to abnormal activity of both the prefrontal cortex [38] and hippocampus [39], which in metformin-treated P301S mice, have an increased burden of inclusions. To investigate whether metformin treatment causes behavioral changes, P301S mice treated with or without metformin were subjected to the OF tasks at 4.5 months of age. At this age, despite extensive brain tauopathy, mice still eat and drink normally, and move freely. Chronic treatment with metformin significantly increased the hyperactive behavior of P301S mice. In metformin-treated P301S mice, travelled distance and speed were significantly higher than those of untreated transgenic mice (Fig. 6d-e). The number of rearings was also significantly higher in metformin-treated than untreated P301S mice (Fig. 6f). When treated with metformin, 5 out of 9 P301S mice also showed abnormal jumping activity (number of jumps / 10 min, 3.3 ± 1.36), which was never observed in untreated P301S mice. The time spent in the center of the arena was similar in all treatment groups (Ctr, 0.1 ± 0.04; Met, 0.1 ± 0.01. *P* = 0.5, Student *t*-test).

Overall, these data indicate that chronic treatment with metformin enhances the hyperactivity typical of P301S mice.

### Metformin treatment exacerbates hindlimb extension reflex deficit in P301S mice

To examine the effects of metformin treatment on neurodegeneration, we evaluated hindlimb extension reflex, which is as an index of motor neuron degeneration [40], throughout the 4-month treatment period. In untreated P301S mice, leg extension progressively deteriorated with age until the final stage, when the hindlimbs were completely retracted with clasped and clenched paws (Fig. 6g). From 18 weeks of age, P301S mice treated with metformin scored significantly worse than untreated transgenic mice (Fig. 6g), indicating that metformin exacerbates hindlimb neurogenic paralysis.

### Metformin treatment induces activation of caspase 3 and cleavage of tau in primary cortical neurons and in the P301S mouse brain

Caspase 3 cleaves tau protein at Asp 421 forming a cleaved-tau (c-Tau) fragment [41, 42], which facilitates tau filament formation in vitro [41, 42] and in vivo [43] and has detrimental effects on synaptic structures [44]. To investigate whether metformin affects caspase 3 mediated-tau cleavage and synapses, we treated primary cortical neurons with metformin for 24 h and examined active, cleaved caspase 3, the levels of its cleavage products c-Tau and cleaved-PARP (c-PARP) and the post-synaptic protein PSD95. In cultured neurons, metformin treatment induces caspase 3 activation, increased levels of c-Tau and c-PARP (Fig. 6h-i) and reduces the expression of post-synaptic density 95 (PSD95; Fig. 6i). Through immunocytochemical analysis, we also found that metformin significantly reduced the number of clusters with overlapping immunoreactivity for the pre-synaptic synapsin I and post-synaptic PSD95 proteins (Fig. 6j), indicating an overall loss of synaptic boutons.

To investigate whether caspase-mediated cleavage of tau occurs in vivo, we analyzed both total and cleaved caspase 3 expression in P301S mice chronically treated with or without metformin. Caspase 3 total protein expression was similar across experimental groups (caspase/tubulin. P301S: Ctr, 0.2 ± 0.06; Met, 0.2 ± 0.03, *P* = 0.6, Student t- test). Consistent with previous findings [18], in untreated P301S mice, cells immunoreactive for active cleaved caspase 3 were never observed in the cortex (Fig. 6k); levels of active cleaved caspase 3 were barely detectable by western blot. After chronic treatment with metformin, some cells were immunoreactive for active caspase 3 in the cortex of P301S mice (Fig. 6k) and cortical levels of active caspase 3 were significantly elevated (Fig. 6l). We next examined c-Tau in the soluble and sarkosyl-insoluble fractions of the cortex of metformin-treated P301S mice. Levels of soluble c-Tau were similar in metformin-treated and untreated P301S mice (Fig. 6m, n). However, in the sarkosyl insoluble fraction, c-Tau levels were significantly higher in metformin-treated P301S mice compared to untreated mice (Fig. 6m, o). To investigate whether chronic metformin affects synapses in vivo, we analyzed the expression of pre- (i.e., synaptophysin and synapsin I) and post-synaptic (i.e., PSD95) proteins by Western blot. Despite levels of synapsin I and PSD95 were unchanged, the expression of synaptophysin was significantly reduced in P301S mice treated with metformin (Fig. 6p-s). Overall, these data suggest that in P301S mice, chronic treatment with metformin induces a small but significant activation of caspase 3, increased caspase-cleaved tau in the tau filament-containing sarkosyl-insoluble cerebral fraction and affects the expression balance of pre-synaptic proteins.

## Discussion

Our findings demonstrate that chronic administration of metformin acts on tau pathology with two distinct mechanisms. First, it reduces tau phosphorylation in the cortex and hippocampus, possibly by inducing PP2A expression via the AMPK/mTOR pathway. Second, it increases the amount of insoluble tau and the number of inclusions with β-sheet aggregates in the brain of P301S mice, likely promoting tau aggregation through direct interaction with the protein. Further, metformin exacerbates hindlimb atrophy, increases P301S hyperactive behavior and induces the activation of caspase 3 with concomitant c-Tau accumulation and synaptic changes.

In AD patients, brain insulin resistance also occurs independently of diabetes or peripheral insulin resistance [6] and is likely a consequence of Aβ pathology [6, 45, 46]. Indeed, Aβ oligomers downregulate insulin receptors in primary cortical neurons [46] and induce insulin resistance in cultured hippocampal neurons [45]. Although insulin resistance markers co-localize with tau inclusions in AD brain [7], the role of tau pathology in the onset of insulin resistance has been unclear: our findings indicate that the two are likely independent. In the P301S mouse, a model of cerebral tauopathy, we find neither peripheral nor central insulin resistance. Indeed, peripheral glucose homeostasis, and brain levels of glucose and insulin are comparable to WT mice and brain insulin signaling is unaffected.

Metformin acts as an insulin sensitizer and enhances glucose utilization in peripheral tissues independently from insulin signaling, via inhibition of mitochondrial complex I and AMPK activation (reviewed in ref. [47]). Metformin has hypoglycemic effects only in the context of diabetes, without affecting glycemia in non-diabetic subjects [48]. Accordingly, we find that chronic metformin affects neither glycemia nor brain insulin signaling in P301S mice, which do not have insulin resistance. Still, metformin has metabolic effects independent of glucose lowering, including induction of a gene expression profile typical of caloric restriction and effects on gut folate microbial metabolism, which have been associated with an increased lifespan in *C. elegans* [49, 50], rats [51] and one, but not another, strain of mice [52]. These findings served as basis to design and propose human studies investigating the potential use of metformin to promote healthy aging [53]. Also, in db/db mice, metformin reduces the phosphorylation of endogenous murine tau without affecting the hyperglycemia induced by insulin resistance [14]. Similarly, in P301S mice, chronic metformin treatment reduces tau phosphorylation on several epitopes, without altering glycemic levels, suggesting a disconnection between the drug’s hypoglycemic action and effects on tau.

Metformin actions on tau phosphorylation may occur via insulin-independent mechanisms, involving AMPK/mTOR and PP2A. It has been previously shown that mTOR plays a key role in modulating tau phosphorylation in vitro and in vivo. Rapamycin reduces tau phosphorylation in primary rodent neurons [17] and in P301S mice [54, 55] and prevents the hyperphosphorylation of tau induced either by high glucose diet [56] or Aβ [57] in the 3XTg-AD mouse. Our results indicate that mTOR inhibition through AMPK is required to activate PP2A and dephosphorylate tau and that this pathway functions independently of insulin.

Tau phosphorylation is also regulated by several kinases. GSK3β plays a key role in tau phosphorylation [24] and is inhibited upon activation of different pathways, including insulin signaling via AKT [58]. In the brain of metformin-treated P301S mice, AKT and insulin signaling are overall unaffected. Accordingly, despite a small trend toward an increase, inhibition of GSK3β is not significantly altered in the cortex of P301S mice treated with metformin, indicating that signaling through this kinase is not involved in the dephosphorylating action of metformin.

Surprisingly, despite reducing tau phosphorylation, chronic metformin increases both the cortical amount of insoluble tau and the number of cells containing inclusions with β-sheet conformation (positively stained by FSB and Gallyas) in the cortex and hippocampus of P301S mice. This is possibly due to different concurring mechanisms. First, our in vitro results indicate that metformin has pro-aggregation effects, leading to the formation of tau aggregates containing β-sheet secondary structure as revealed by thioflavin T binding. The thioflavin T signal of aggregates induced by metformin is lower than that of filaments obtained with heparin. This is possibly due to different ultrastructural features. In the presence of heparin, tau aggregates in long filaments, while metformin-induced aggregates appear as tau protofibrils made of globular oligomers. Consistently, Western blot analysis of sarkosyl-insoluble tau reveals that in the cortex of P301S mice treated with metformin there is an increased amount of oligomeric species, mainly dimers. Metformin-induced globular aggregates resemble SNUs, which form during the initial stages of heparin-induced aggregation in vitro and likely represent tau oligomers or precursors of tau filaments [37]. It has been shown that SNUs bear β-sheet secondary structure [59]. However, the β-sheet content is somehow lower than that of heparin-induced aggregates and is associated with reduced thioflavin T signal [59]. Within this context, our results suggest that chronic exposure to metformin may catalyze the aggregation of tau by promoting the formation of oligomeric species or SNUs.

An alternate mechanism by which metformin may increase the amount of insoluble tau filamentous pathology in vivo is through caspase-mediated cleavage of tau. Caspase 3 cleaves tau at Asp421, forming a truncated form of tau, which aggregates readily and triggers the assembly of full-length tau into insoluble fragments [41]. Previous studies demonstrated that caspase 3-mediated cleavage of tau is an early event in AD tau pathology [42]. Caspase 3 activation is induced by appoptosin overexpression, which is increased in the brain of patients with AD and frontotemporal dementia with tau inclusions [44]. In living Tg4510 tau transgenic mice, caspase 3 activation precedes and leads to tau inclusions formation [43]. Our findings indicate that in P301S tau transgenic mice, chronic metformin enhances this pathological process by inducing activation of caspase 3 and accumulation of c-Tau in the cerebral fraction containing insoluble tau filaments.

Among biguanides, only metformin displays a dual action on tauopathy, i.e. dephosphorylating and pro-aggregating. The butyl-substituted biguanide, buformin, retains some pro-aggregating properties, but has no significant effects on tau phosphorylation in vitro. Conversely, phenformin, which bears a phenyl-ethyl side chain, has no pro-aggregating property in vitro, but maintains tau dephosphorylating properties. It is unclear how metformin and buformin act on tau aggregation. Being positively charged at physiological pH, it is plausible that these molecules electrostatically interact with tau, neutralizing negative charges and promoting aggregation. Further studies are warranted to clarify this fundamental point.

The potential benefits of metformin on cognition and AD are still controversial. In rodents, metformin significantly attenuated the cognitive impairment induced by insulin resistance and metabolic dysfunction as tested with the Morris water maze [12], but has no effect on other cognitive tasks, such as behavioral flexibility [13] or fear conditioning [14]. Still, metformin is neuroprotective in a mouse model of Huntington disease [60] and improves cognitive function in experimental models unrelated to metabolic dysfunction such as the pentylentetrazole-induced kindling [15] and the haloperidol-induced catalepsy in adult mice [16]. In contrast, other studies point to potential adverse effects of this drug on cognitive performance and AD. For example, metformin has been associated with impaired cognitive performance in elderly patients (>75 years of age) with diabetes [61]. Further, diabetic patients taking metformin have an increased risk of developing AD than those taking other antidiabetic drugs [62]. Lastly, metformin increases the generation of Aβ in cultured neurons due to induction of BACE expression [63]. These findings together with our observation that metformin may promote tau aggregation in vivo raise concerns on the potential overall benefits of metformin for AD and require further investigation in the elderly population.

Previous intervention studies with drugs targeting tau pathology report that a parallel decrease in both phosphorylation and aggregation of tau improves behavioral outcomes in the P301S mouse [54, 64]. Our results instead indicate that metformin’s opposing actions on tau phosphorylation and aggregation worsens hindlimb atrophy and the hyperactive behavior of P301S mice. Such behavioral outcomes suggest that in AD, metformin may negatively impact on agitation, which is one of the most disturbing neuropsychiatric symptoms of AD [65]. However, the behavioral phenotypes evaluated in our study are unrelated to cognitive changes associated with AD. Unfortunately, the strong motor impairment of the P301S transgenic mouse limits the possibility of investigating the effects of tauopathy on cognitive domains. Thus, further studies are warranted to fully clarify the role of tau aggregates and/or potential tau-unrelated actions of metformin on cognition in other transgenic models of tauopathy with cognitive deficits and normal motor function, such as the Tg4510 mouse [66].

## Conclusions

Our findings indicate that metformin pro-aggregation effects mitigate the potential benefits arising from its dephosphorylating action. Metformin is the most widely prescribed antidiabetic drug, accounting for about 50 % of prescriptions for non-insulin antidiabetic drugs in 2012 [67]. In the elderly population, there is a high degree of AD and T2DM comorbidity due to the high incidence of these pathological conditions during aging [68, 69] and T2DM patients have an increased risk of developing AD [3, 4]. Due to its dual actions on tau phosphorylation and aggregation, in chronic administration regimens, metformin may unpredictably impact the development of tauopathy with undefined outcomes in elderly diabetic patients at risk for AD. Our results indicate that the effects of metformin on tau pathology manifest at doses yielding serum concentrations consistent with those of therapeutic dosing in humans [22]. Given the projected increase of prevalence of both diabetes and AD over the next decades [68, 69], further studies are warranted to investigate the effects of metformin on AD development, its contribution to the increased risk of AD associated with T2DM and its therapeutic potential for tau-related neurodegenerative conditions. Conversely, from a long-term perspective, our findings indicate that the effects of metformin on tau phosphorylation and aggregation could be dissociated through chemical modifications and point to AMPK/mTOR signaling as a potential druggable target for tauopathy.

## Methods

### Antibodies and reagents

The following primary antibodies were used: mouse monoclonal antibodies against GSK3β (Millipore), IRβ, S6 (Santa Cruz), βI-tubulin (Sigma), β-actin (Sigma), caspase cleaved tau (Millipore), c-PARP, AKT (pan), IRS1 (Cell Signaling), PSD95 (Cell Signaling for Western blot or Millipore for immunocytochemistry), Synapsin-I (Millipore), GFP (Roche) and Rheb (Santa Cruz Biotechnologies); rabbit polyclonal antisera against p[Ser1162/1165]IRβ, p[S9]GSK3β, AMPK, p[T172]AMPK, PP2A, p[S240/244]S6, mTOR, p[S2448]mTOR, p[S473]AKT, active cleaved caspase 3 (Cell Signaling), actin (Sigma) and p[S616]IRS1 (Invitrogen), Synaptophysin (Cell Signaling). For total tau and phospho-tau analysis, we used the monoclonal antibodies MC1 [36] and PHF1 [70] (kind gift of Dr P.Davies, Albert Einstein College of Medicine, New York, NY), AT100, AT270 (Autogen Bioclear), Tau5, AT8 (Calbiochem) and the polyclonal anti-p[S262]Tau antibody (Invitrogen). Secondary antibodies conjugated with Alexa fluorophores were from Invitrogen. For western blot analysis, horseradish-peroxidase (HRP)-conjugated secondary antibodies (Thermo Scientific) or ECL Plex Cy3-conjugated goat anti-mouse IgG and Cy5-conjugated goat anti-rabbit IgG (GE Healthcare) were used.

Stock solutions of metformin, phenformin (Sigma), buformin (Santa Cruz) were prepared in water. Insulin (Humulin R® U-500, 100 UI/ml, Eli Lilly), heparin (12–15 kDa, 5000 UI/ml, Sirton Pharmaceuticals), rapamycin (LC labs; 25 mM in Ethanol), dorsomorphin (25 mM in DMSO), and okadaic acid (Sigma; 250 μM in DMSO) were used for experiments in vitro. Unless otherwise specified, general reagents and chemicals were from Sigma and cell culture reagents were from Invitrogen.

### Animals and pharmacological treatments

Male homozygous P301S transgenic [18] and age-matched C57/Bl6 wild type (WT) non-transgenic mice were used. Animal health and comfort were veterinary-controlled. Mice were housed in filtered cages in a temperature-controlled room with a 12:12 h dark/light cycle with ad libitum access to water and food. All animal experiments were performed in full compliance with the European Community Council directive dated 86/609/EEC, the revised directive 2010/63/EU and were approved by the Italian Ministry of Health and by IIT Animal Facility Committee. At 4 weeks of age, P301S were randomly assigned to treatment or control groups (12–15 mice/group). Animals in the treatment group received 2 mg/ml metformin in the drinking water for 4 months as previously described [60]. Drinking bottles were replenished with fresh water or metformin solution every week. The dose of metformin was equivalent to ≈ 300 mg/kg/day and was selected based on pharmacokinetic studies of metformin distribution in rodents [71] and initial pharmacokinetic analyses in WT and P301S mice treated for 7 days with metformin. In rats, chronic administration of such dose achieves plasma levels (7.6 μmol/l) [71] comparable to those obtained by therapeutic dosing in humans (7.8 ÷ 23.3 μmol/l) [22] and yields similar levels in the frontal cortex (4.3 ± 1.0 nmol/g of tissue) and hippocampus (4.3 ± 0.7 nmol/g) [71]. Using HPLC-MS analysis as in [71], we measured the concentration of metformin in plasma and brain of WT and P301S mice treated for 7 days. Metformin concentration in plasma was in the low micromolar range and was similar in WT and P301S mice treated for 7 days (Additional file 1: Table S1). Levels of metformin in the brain were also comparable in WT and P301S mice (Additional file 1: Table S1). In untreated WT and P301S mice, metformin was undetectable in plasma and brain. Levels of metformin were also determined in the plasma of P301S mice treated for 4 months. After chronic treatment, plasma levels of metformin (μmol/l ± SD, 0.7 ± 0.4, *n* = 6) were similar to those achieved by 7 day administration (Additional file 1: Table S1). During chronic administration with metformin, body weight, glycemia, water and food consumption were monitored weekly. Glycemia, water and food consumption were unaffected by metformin (Additional file 1: Figure S4). In P301S mice, body weight steadily increased from 1 to 4 months of age. From 4 months of age, the body weight stops increasing (Additional file 1: Figure S4A), because of progressive muscle atrophy [18]. Metformin treatment did not significantly influence body weight.

### Intraperitoneal glucose tolerance test and insulin tolerance test

For IPGTT, after fasting overnight for 16 h, 5-month old mice were challenged by intraperitoneal injection of 2 g/kg glucose bolus. For ITT, mice were adapted by intraperitoneal saline solution injection for 3 days. After fasting for 6 h, mice were injected intraperitoneally with 0.5 UI/kg insulin. Blood samples were drawn from tail nicks over a 2 h period at selected time intervals. Blood glucose content was measured by using GlucoCards and the GlucoCard G Meter (A.Menarini), following the manufacturer’s instructions.

### Glucose and insulin assays

Glucose was measured using the Amplex red glucose assay kit (Invitrogen) following the manufacturer’s instructions. Mouse hemi-cortices were lysed in 20 mM Tris HCl pH 7.5, 150 mM NaCl 1 mM EDTA, 1 mM EGTA, 1 % Triton X-100, and phosphatases and proteases inhibitors by trituration using a glass-glass dounce tissue homogenizer on ice. Lysates were spun at 30,000 x g using a Beckman Optima Ultra Centrifuge and the MLA 130 Rotor for 30 min at 4 °C. The supernatant was collected for glucose measurement with Amplex red. The fluorescence values were read at 545 nm excitation and 590 nm emission wavelengths using a Victor 3 Multi-label Microplate Reader (Perkin Elmer).

Cortical insulin content was measured using a specific sandwich ELISA for mouse insulin (Millipore) following the manufacturer’s instructions.

### Behavioral tests

#### Hind limb extension test

The hind limb extension test [40] was performed weekly for the entire treatment period. Mice were held at the tip of the tail and suspended 30 cm above the floor for 20 s. Mice were scored from 0–5 based on the degree of hindlimb extension reflex or the presence of paw clasping. An animal received a score of 0 when the leg extension reflex was normal. Score 1 and 2 were assigned when mice retracted the hindlimbs after 15 or 5 s, respectively. Score 3 and 4 were given when mice showed asymmetric retraction of one leg (score 3) or both legs (score 4) from the beginning of the test. Mice scored 5 when hindlimbs were completely retracted towards the body and paws were clasped for the entire test time.

#### Open-field

The open field protocol was adapted from [20]. Briefly, at 17–18 weeks of age, all mice underwent a single 20 min trial session of open-field testing. Mice were first acclimated to the room for 15 min the same day of testing. To start the test, each animal was placed facing the wall in an open-field arena (44 cm L × 44 cm W × 44 cm H) made of black plexiglass, with a light grey bottom. Behavioral performance was recorded using a video tracking system (Any-Maze; Ugo Basile, Varese, Italy). Behavioral analyses were carried out on recorded movies using the AnyMaze software (Stoelting). The same observer, who was unaware of the animals’ genotype, assigned all scores. The 20-min trial session was divided into four blocks (sessions) of 5 min each. During the 20-min session, the frequency of the following behavioral parameters was scored: traveled distance, speed, time in the center, and jumping (only frequency). During the central 10 min test phase (2^nd^-3^rd^ 5-min sessions), the following behavioral parameters were scored: wall rearing, standing on the hindlimbs and touching the walls of the apparatus with the forelimbs; rearing, standing on the hindlimbs in the open (away from the walls of the apparatus); grooming, licking and mouthing its own fur, sometimes with the help of the forepaws.

### Primary neuronal cultures and pharmacological treatment

Primary cortical neurons were isolated from the brain of WT embryos of either sex at E18 as previously described [72]. Embryonic cortices were collected in DMEM, and cells were dissociated by incubation with trypsin/EDTA at 37 °C. Cells were then diluted in neurobasal medium containing B27 supplement, Glutamax, penicillin/streptomycin and plated at a density of 7.0 × 10^5^ cells/well on 6-well plates coated with 0.1 mg/ml poly-L-lysine (PLL; Sigma). Neurons were cultured at 37 °C in a humidified incubator with 5 % CO_2_. After 6 days, the medium was replaced with medium containing B27 without insulin. Twenty-four hours later, neurons were incubated with or without 2.5 mM metformin, 30 μM phenformin, 30 μM buformin, 10 μM rapamycin, 10 μM dorsomorphin and/or 10 nM okadaic acid for 6 h. Alternatively, neurons were incubated with or without 0.5 mM metformin for 24 h as indicated in selected experiments. In selected experiments, neurons were maintained in medium containing B27 with insulin for the entire treatment period. After pharmacological treatments, neuronal viability was assessed using the MTT assay as described elsewhere [72]. None of the pharmacological treatments altered the viability of neurons over 6 h (Additional file 1: Table S2).

### Cortical neurons transduction with lentiviral particles

Lentiviral plasmids pHAGE-CMV-Rheb(S16H)-IRES-eGFP-W [73] and pHAGE-CMV-IRES-eGFP-W [74] were obtained by Addgene and verified by DNA sequencing. Lentiviral particles were produced according to [75]. Neurons were plated at a density of 5 × 10^5^ /well onto PLL-coated 6-well plates, incubated at 37 °C with 5 % CO_2_ for 4 days and then transduced with 10 MOI of lentiviral particles for 24 h. Six days after infection, the medium was replaced and after 24 h neurons were treated for 6 h with 2.5 mM metformin.

### Western blots

Brain tissues or primary cortical neurons were sonicated in 150 mM NaCl, 10 mM Tris, pH 7.4, 1 mM EGTA, 0.5 % Triton X-100, and protease and phosphatase inhibitors and incubated for 30 min on ice. Samples were spun at 20,800 x g in an Eppendorf 5417R centrifuge for 45 min. Supernatants were collected and protein concentration was determined using the BCA kit (Pierce).

Equal amount of proteins were separated by electrophoresis on 10 % SDS-PAGE as previously described [76] and transferred overnight at 4 °C onto Protran 0.2 NC nitrocellulose membranes (Amersham). Membranes were blocked for 1 h at room temperature with 5 % nonfat milk or bovine serum albumin (BSA) in blocking buffer containing 0.05 % Tween-20 in phosphate buffered saline (PBS) pH 7.4 and incubated overnight at 4 °C with primary antibodies. Membranes were rinsed in Tris buffered saline pH 7.4 with 0.05 % Tween 20 (TBST) and incubated for 1 h at room temperature with secondary antibodies. Chemiluminescent signals were visualized using the SuperSignal West Pico Chemiluminescent Substrate (Thermo Scientific) and acquired using Image Quant LAS 4000 mini (GE Healthcare). Fluorescent signals were acquired using Typhoon Trio + and Typhoon Scanner Control v5.0 software (GE Healthcare). Densitometric analysis was performed using ImageJ software [77]. Protein levels were normalized on βI-tubulin or actin. Phosphorylated proteins were normalized to the respective total protein. Data were expressed as percentage of WT untreated mice unless otherwise specified.

### RNA isolation and quantitative real time PCR (qRT-PCR)

RNA was isolated using the Qiazol extraction protocol with 5 PRIME Phase lock Gel Tubes (Fisher Scientific) according to the manufacturer’s protocol. RNA was quantified using an ND1000 Nanodrop spectrophotometer (NanoDrop Technologies, Inc). Reverse transcription of 1 μg of RNA was performed using the RT2 First Strand Kit (Qiagen, Hilden, Germany) according to the manufacturer’s instructions. qRT-PCR was performed using the QuantiFast SYBR Green PCR Kit (Qiagen) on a 7900HT Real-time System Instrument equipped with Sequence Detection Systems 2.3 Software (Applied Biosystem) as previously described [72]. Data were normalized to the expression of multiple housekeeping genes, including actin, GAPDH and HPRT1, as described [72], and Ct values were converted into fold-expression values relative to control using qBase^PLUS^ software (Biogazelle). Primers are listed in Additional file 1: Table S3.

### Immunocytochemistry

Neurons were fixed in 4 % paraformaldehyde (PFA), permeabilized, blocked in 5 % BSA and then incubated overnight at 4 °C with anti-synapsin-I and PSD95 antibodies in BSA. Specimens were visualized under a Decon microscope (Axio Observer Z1, ZEISS).

### Immunohistochemistry and confocal microscopy

Mice were deeply anesthetized with 20 % urethane in saline and transcardially perfused with saline followed by 4 % PFA in PBS. Brains were then dissected, post-fixed in 4 % PFA for 24 h at 4 °C and cryopreserved in 30 % sucrose in PBS for 24 h. 30 μm sections were cut using a Microm HM450 sliding microtome (BioOptica). Immunohistochemical detection of tau and phospho-tau was performed as described [76] using sagittal sections collected from −0.48 to −1.32 mm from bregma. Insoluble aggregates with β-sheet conformation were detected by Gallyas silver staining or staining with the Congo red analogue FSB (DojinDo Laboratories) [78] as previously described [76]. Hoechst-33342 (Sigma) was used to stain nuclei. For cell counts, fluorescence images of entire sections were collected using an automated Olympus BX51 microscope, equipped with a MBF Optonic CX9000 camera, UPLFLN SEMIAPO FLUORITE 10X NA objective and MBF Neurolucida V11 software. An experienced researcher blind to the nature of the samples counted the numbers of AT8-, Gallyas- and FSB-positive cells. 3–5 slices were analyzed per animal. Cell counts were normalized to the area of the respective brain region.

Confocal optical sectioning was performed with 1 μm step using an inverted Confocal Leica TCS SP5 AOBS TANDEM Leica Las AF equipped with an HCX PL APO 40X 1.25 NA oil objective.

### Tau purification and aggregation

Recombinant tau was produced as described previously [79]. Briefly, the cDNA of the 441-amino acid isoform of human brain tau (2 N, 4R) was cloned in the pET-21 plasmid and expressed in *Escherichia coli* BL21 (DE3). Cultures were grown to mid-log phase and isopropyl-1-thio-β-D-galactopyranoside was added to 400 μM. After 4 h shaking at 220 rpm at 30 °C, cells were pelleted, lysed in 50 mM PIPES pH 6.8 by boiling for 5 min, placed on ice, and 1 mM DTT was added. The lysate was spun at 38,000 x g for 1 h at 4 °C. The supernatant was collected, subjected to ion exchange chromatography using HITRAP CM FF (GE Healthcare), eluted in 50 mM PIPES pH 6.8 and a gradient of NaCl (up to 0.5 M). Fractions were run on a 10 % Tris-glycine gel and stained with Comassie to examine recombinant tau. Tau-containing fractions were pooled, chromatographed on a Superdex-75 size exclusion column (GE Healthcare) and eluted in 25 mM Tris-HCl, pH 7.4. The absorbance at 220 and 280 nm was measured to determine the concentration of the freshly purified protein. Purified recombinant protein stocks were stored in aliquots at −80 °C until use.

Purified recombinant tau (50 μM), 37.5 μM heparin (molar ratio tau/heparin = 0.75) and/or 30 μM metformin, buformin or phenformin were incubated at 37 °C for 8–15 days in PBS pH 7.4, containing 1 mM EDTA and 1 mM EGTA. Tau aggregation was monitored using the Thioflavin T (ThT; Sigma) assay. Sample fluorescence was examined using a Tecan Infinite plate reader set at 430 nm for excitation and 485 nm for emission. Tau aggregates were also analyzed by western blot and transmission electron microscopy.

### Sarkosyl extraction

Sarkosyl-insoluble tau was extracted as described previously [76]. Briefly, tissues were homogenized in 10 volumes of cold extraction buffer (10 mM Tris-HCl, pH 7.4, 0.8 M NaCl, 1 mM EGTA, 10 % sucrose) and the homogenates were spun for 30 min at 20,000 × *g*. After adding 1 % sarkosyl, the samples were shaken for 1 h at room temperature and spun at 100,000 × *g* for 1 h *at* 4 °C. The pellets containing insoluble tau were resuspended in 50 mM Tris-HCl, pH 7.4 and stored at 4 °C until analysis by western blot.

### Negative staining transmission electron microscopy

Recombinant tau protein aggregates were placed onto 300-mesh glow discharged carbon-coated grids and negatively stained in 2 % uranyl acetate. The samples were observed with a FEI Tecnai F20 field emission gun transmission electron microscope (FEI Company, The Netherlands), working at an acceleration voltage of 80 keV and equipped with a 2k X 2K Gatan Ultrascan CCD camera (Gatan company, USA).

### Statistical analysis

Statistical analysis of groups with normal distributions was performed using the Student’s *t*-test for two groups or ANOVA for multiple comparisons. One-way ANOVA or two-way ANOVA followed by multiple comparisons with the Holm-Sidak method was performed for more than two groups. Differences among groups were considered statistically significant when p < 0.05. Data throughout the text are reported as average values ± SEM unless otherwise specified.

## Additional file

Additional file 1: Figure S1. Expression levels of key molecules in the insulin pathway in WT and P301S mouse cortex. **Figure S2.** Expression levels of key molecules in the insulin pathway in the cortex of P301S mouse treated with or without metformin. **Figure S3.** Expression levels of AMPK, S6, pS6, and tau in primary cortical neurons treated with or without metformin and/or specific blockers. **Figure S4.** Body weight, glycemia, water and food consumption in P301S mice treated with or without metformin. **Table S1.** Concentration of metformin in the plasma and brain of WT and P301S mice after treatment for 7 days. **Table S2.** Neuronal viability after pharmacological treatment for 6 h. **Table S3.** Primers used for qRT-PCR. (PDF 5110 kb)

## Abbreviations

AD: Alzheimer disease
AKT: protein kinase B
AMPK: AMP-activated protein kinase
AUC: area under the curve
Aβ: β-amyloid
BSA: bovine serum albumin
Buf: buformin
c-caspase 3: cleaved caspase 3
cDNA: coding DNA
c-PARP: cleaved PARP
CSF: cerebrospinal fluid
c-Tau: cleaved tau
Ctr: control
Dorso: dorsomorphin
GSK3β: glycogen synthase 3β
Hep: heparin
IPGTT: intraperitoneal glucose tolerance test
IR: insulin receptor
IRS-1: IR scaffold-1
IRβ: IR β subunit
ITT: insulin tolerance test
Met: metformin
mTOR: mammalian target of rapamycyn
OA: okadaic acid
OF: open field
P301S: P301S mutant human tau transgenic mouse
pAKT: phospho-AKT
pAMPK: phospho-AMPK
PBS: phosphate buffered saline
PFA: paraformaldehyde
pGSK3β: phospho-GSK3β
Phe: phenformin
PP2A: protein phosphatase 2A
PSD95: post-synaptic density 95
pTau: phospho-tau
qRT-PCR: quantitative real time polymerase chain reaction
Rapa: rapamycin
SNUs: spherical nucleation units
T2DM: type 2 diabetes mellitus
TEM: transmission electron microscopy
ThT: thioflavin T
WT: wild type
