# Supplementary material for: Metformin promotes tau aggregation and exacerbates abnormal behavior in a mouse model of tauopathy
Source: Mol Neurodegener. 2016 Feb 9;11:16. doi: 10.1186/s13024-016-0082-7 (PMC4746897; doi:10.1186/s13024-016-0082-7)
Supplement: Additional file 1: Figure S1. — Expression levels of key molecules in the insulin pathway in WT and P301S mouse cortex. Figure S2. Expression levels of key molecules in the insulin pathway in the cortex of P301S mouse treated with or without metformin. Figure S3. Expression levels of AMPK, S6, pS6, and tau in primary cortical neurons treated with or without metformin and/or specific blockers. Figure S4. Body weight, glycemia, water and food consumption in P301S mice treated with or without metformin. Table S1. Concentration of metformin in the plasma and brain of WT and P301S mice after treatment for 7 days. Table S2. Neuronal viability after pharmacological treatment for 6 h. Table S3. Primers used for qRT-PCR. (PDF 5110 kb) [file 13024_2016_82_MOESM1_ESM.pdf]

Metformin promotes tau aggregation and exacerbates abnormal behavior in a mouse model of tauopathy

Erica Barini[1]^, Odetta Antico[1]^, Yingjun Zhao[2,3], Francesco Asta[1], Valter Tucci[1], Tiziano Catelani[4], Roberto Marotta[4], Huaxi Xu[2,3], Laura Gasparini[1,*]

[1]Dept. of Neuroscience and Brain Technologies, Istituto Italiano di Tecnologia, Via Morego 30, Genova, Italy; [2]Fujian Provincial Key Laboratory of Neurodegenerative Disease and Aging Research, Institute of Neuroscience, College of Medicine, Xiamen University, Xiamen, Fujian 361102, China; [3]Degenerative Diseases Program, Sanford Burnham Prebys Medical Discovery Institute, La Jolla, CA 92037, USA; [4]Electron Microscopy Lab, Nanochemistry Department, Istituto Italiano di Tecnologia, Via Morego 30, Genova, Italy.

^equal contribution.

Supplementary Materials:

Figure S1 - Expression levels of key molecules in the insulin pathway in WT and P301S mouse cortex

Figure S2 - Expression levels of key molecules in the insulin pathway in the cortex of P301S mouse treated with or without metformin

Figure S3 - Expression levels of AMPK, S6, pS6, and tau in primary cortical neurons treated with or without metformin and/or specific blockers

Figure S4 - Body weight, glycemia, water and food consumption in P301S mice treated with or without metformin

Table S1 - Concentration of metformin in the plasma and brain of WT and P301S mice after treatment for 7 days

Table S2 – Neuronal viability after pharmacological treatment for 6 h

Table S3 – Primers used for qRT-PCR

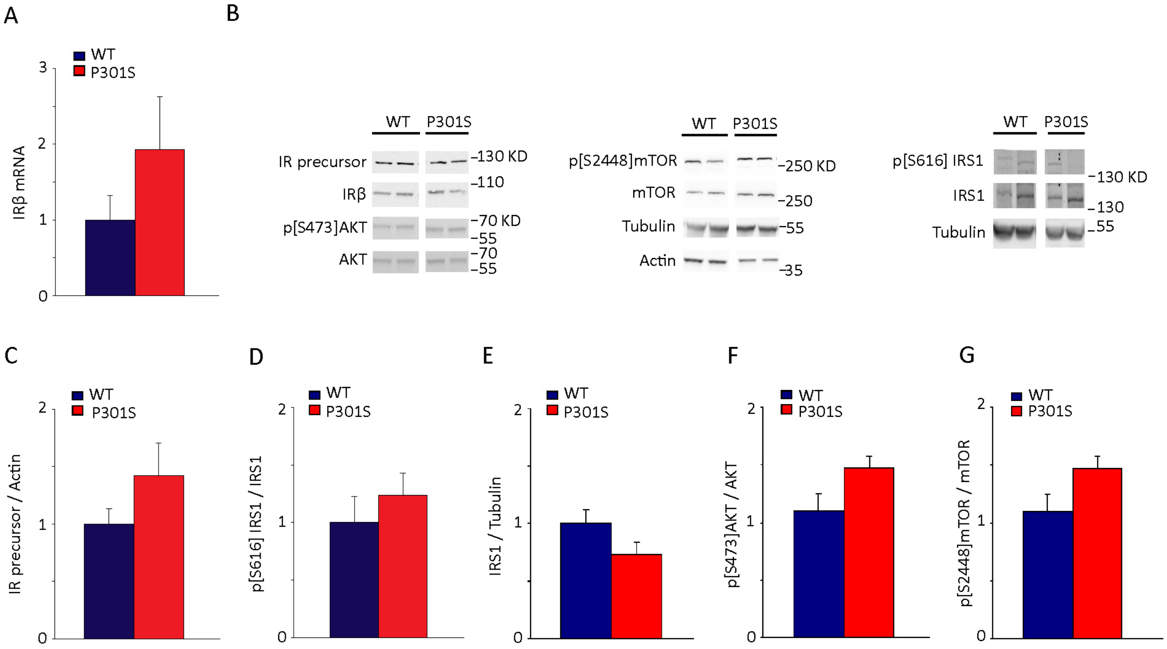

Figure S1 – Expression levels of key molecules in the insulin pathway in WT and P301S mouse cortex

A. IRβ gene expression in the cortex of 5-month old P301S and WT mice. Data are presented as average fold expression over WT ± SEM. **B.** Western blot of mTOR, IRβ precursor, IRβ, Akt, p[S473]AKT, mTOR, p[S2448]mTOR, IRS1 and p[S616]IRS1 in the cortex. Actin and Tubulin were analyzed as a loading control. **C-G.** Quantitative analysis of IRβ precursor (C), p[S616]-IRS1 (D), total IRS-1 (E), p[S473]AKT (F), and p[S2448]mTOR (G) protein expression in the cortex of WT and P301S mice. Total protein expression levels were normalized to actin or tubulin as indicated. Levels of phosphorylated proteins were normalized to the total respective protein. Bars represent the average ratio ± SEM. Numbers of animals per group were as follows. Panel A: WT, n = 6; P301S, n = 5. Panel B: n = 7 / group. Panels C and D: WT, n = 5; P301S, n = 7. Panels E: WT, n = 8; P301S, n = 12. Panels E: n = 7 / group.

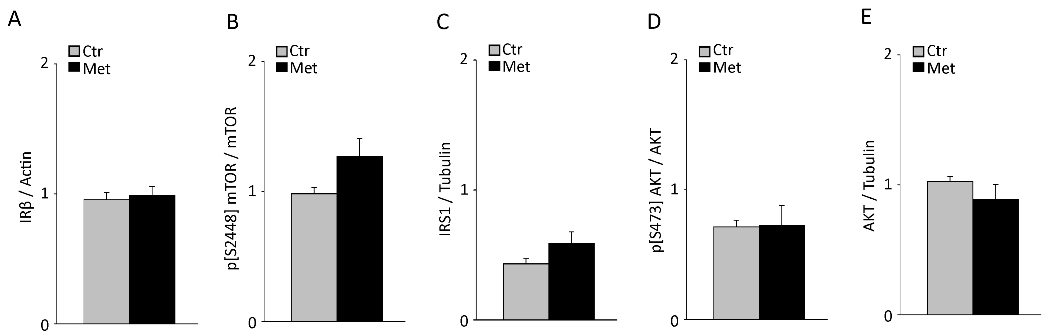

Figure S2 - Expression levels of key molecules in the insulin pathway in the cortex of P301S mouse treated with or without metformin

P301S were treated with metformin (Met) or untreated (Ctr) for 4 months starting at 1 month of age. Cortical protein levels of IRβ, mTOR phosphorylated on Ser2448 (p[S2448]mTOR), IRS1, AKT and its activated form phosphorylated on Ser472 (p[S473]AKT) were analyzed by western blot. **A-E.** Quantitative analysis of IRβ (A), p[S2448]mTOR (B), IRS1 (C), p[S473]AKT (D) and AKT (E) in the cortex. Protein levels were normalized to actin or tubulin. Levels of phosphorylated proteins were normalized to the total respective protein. Bars represent the average ratio ± SEM. In all graphs, n = 12 / group.

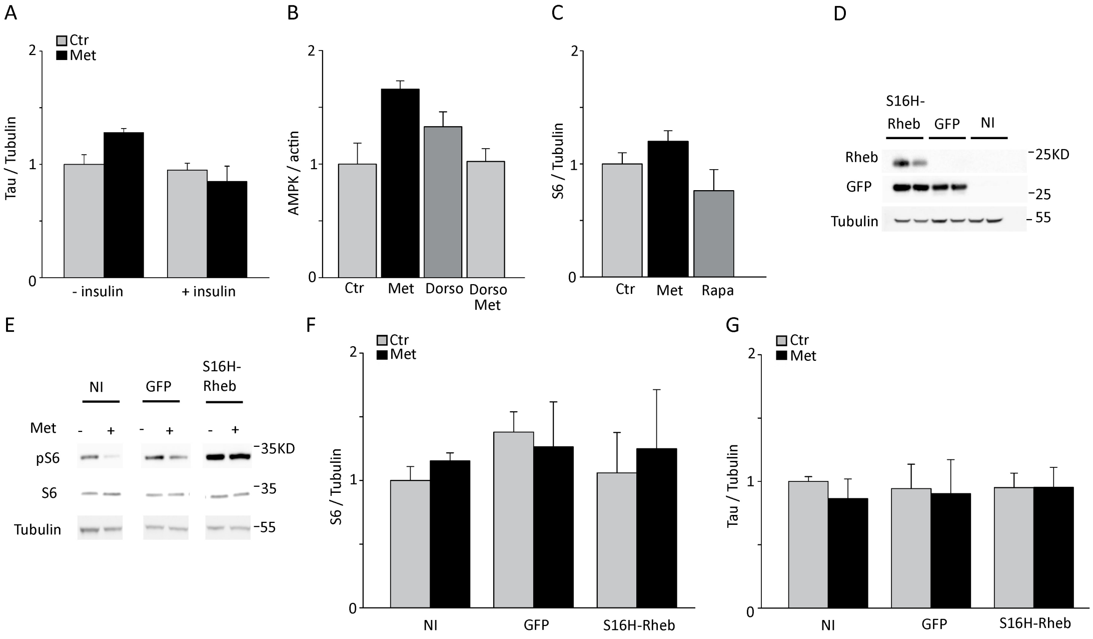

Figure S3 - Expression levels of AMPK, S6, pS6 and tau in primary cortical neurons treated with or without metformin and/or specific blockers

Primary WT cortical neurons were incubated for 6 h in 2.5 µM metformin (Met), 10 µM rapamycin (Rapa), 10 µM dorsomorphin (Dorso) alone or in combination. Levels of tau, AMPK, S6 and phospho-S6 (pS6) were analyzed by western blot. **A**. Quantitative analysis of total tau in cortical neurons treated with or without metformin in the absence or presence of insulin. **B.** Quantitative analysis of total AMPK in neurons treated with metformin and dorsomorphin alone or in combination. **C**. Quantitative analysis of S6 in neurons incubated with metformin or rapamycin in the absence of insulin. **D-E.** Primary cortical neurons were transduced with S16H-Rheb and GFP, GFP control or not infected (NI) and treated with or without metformin for 6 h. **D.** Western blot analysis of Rheb, GFP and tubulin in transduced neurons. **E.** Western blot analysis of pS6, S6 and tubulin in transduced neurons treated with or without metformin. **F-G.** Quantitative analysis of S6 (F) and total tau (G) expression in transduced neurons. Total tau was detected using Tau5 antibody.

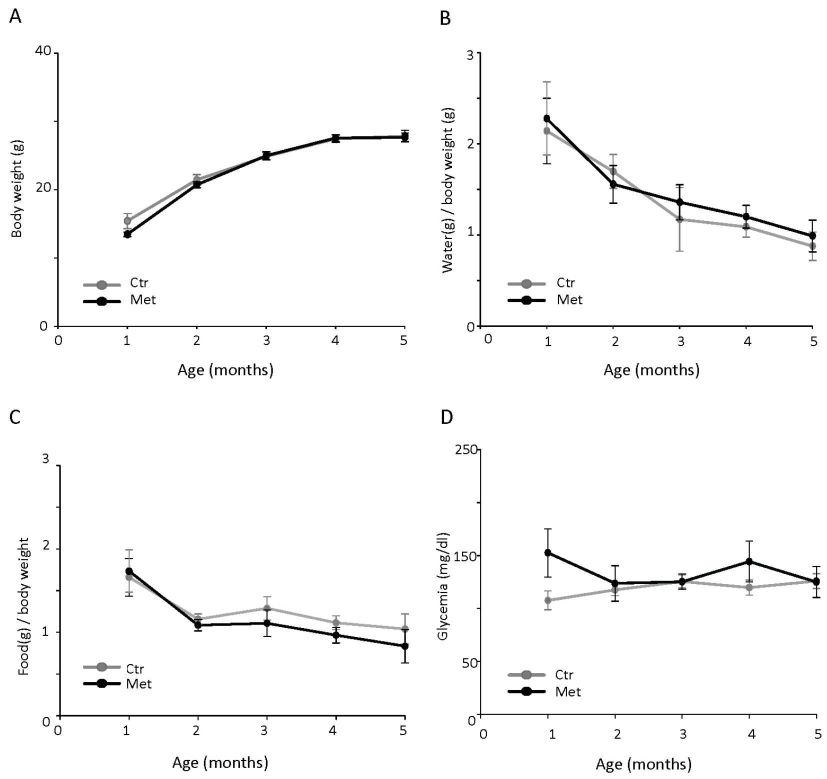

Figure S4 – Body weight, glycemia, water and food consumption in P301S mice treated with or without metformin

P301S mice were treated with metformin (Met ; 2 mg/ml in the drinking water) for 4 months starting at 1 month of age. The control group (Ctr) received regular drinking water. **A.** Body weight. **B**. Water consumption. **C.** Food consumption. **D.** Glycemia. Values of water and food consumption were normalized to body weight. Data points represent average ± SEM. Numbers of animals per group were as follows. Ctr, n = 12; Met, n = 13.

Table S1 – Concentration of metformin in the plasma and brain of WT and P301S mice after treatment for 7 days.

	**Plasma** µmol/l ± SD [n]	**Brain** nmol/g tissue ± SD [n]
WT	1.3 ± 0.4 [6]	0.25 ± 0.08 [9]
P301S	0.9 ± 0.7 [7]	0.19 ± 0.08 [9]

[n], number of animals.

Table S2 – Neuronal viability after pharmacological treatment for 6 h

Drug	Final concentration	Viability ± SEM	P value[#]
Control	-	1.0±0.05	-
Vehicle (DMSO)	0.000001 %	1.04±0.2	0.1[a]
Vehicle (Ethanol)	0.005 %	1.1±0.1	0.4[a]
Metformin	2.5 mM	1.1±0.05	0.2[a]
Buformin	30 µM	0.9±0.03	0.2[a]
Phenformin	30 µM	0.97±0.05	0.8[a]
Dorsomorphin	10 µM	0.8±0.04	0.4[c]
Rapamycin	10 µM	1.1±0.05	0.8[b]
Okadaic Acid	10 nM	0.8±0.06	0.5[c]

Neuronal viability was determined by the MTT assay. [#]Student t-test versus control[a] or vehicle (Ethanol[b] or DMSO[c]).

Table S3 – Primers used for qRT-PCR

Gene	Forward	Reverse	Efficiency
PP2A	5’-GTTCGGTTCCTCCTCTGT -3’	5’- GCTGTCCTGCAACAACTT -3’	1.85
AMPK	5’GGCTCTGACATGATGAATGGAATG -3’	5’-AGGTTACTCTGGGCAAACATACA -3’	1.87
actin	5’-AAGTGGTTACAGGAAGTCC-3’	5’-ATAATTTACACAGAAGCAATGC-3’	2.08
GAPDH	5’-GAACATCATCCCTGCATCCA-3’	5’-CCAGTGAGCTTCCCGTTCA-3’	1.98
HPRT1	5’- CGAGGAGTCCTGTTGATGTTGC -3’	5’- CTGGCCTATAGGCTCATAGTGC-3’	2.08
